# Loss of β-catenin via activated GSK3β causes diabetic retinal neurodegeneration by instigating a vicious cycle of oxidative stress-driven mitochondrial impairment

**DOI:** 10.18632/aging.103446

**Published:** 2020-06-23

**Authors:** Xing-Sheng Shu, Huazhang Zhu, Xiaoyan Huang, Yangfan Yang, Dandan Wang, Yiling Zhang, Weizhen Zhang, Ying Ying

**Affiliations:** 1 Department of Physiology, School of Basic Medical Sciences, Shenzhen University Health Sciences Center, Shenzhen, Guangdong, China; 2 State Key Laboratory of Ophthalmology, Zhongshan Ophthalmic Center, Sun Yat-Sen University, Guangzhou, Guangdong, China; 3 Department of Medicine, Li Ka Shing Faculty of Medicine, The University of Hong Kong, Pokfulam, Hong Kong, China; 4 Department of Physiology and Pathophysiology, Peking University Health Science Center, Beijing, China

**Keywords:** GSK3β/β-catenin signaling, oxidative stress-driven mitochondrial impairment, diabetic retinal neurodegeneration

## Abstract

Synaptic neurodegeneration of retinal ganglion cells (RGCs) is the earliest event in the pathogenesis of diabetic retinopathy. Our previous study proposed that impairment of mitochondrial trafficking by hyperphosphorylated tau is a potential contributor to RGCs synapse degeneration. However, other molecular mechanisms underlying mitochondrial defect in diabetic retinal neurodegeneration remain to be elucidated. Here, using a high-fat diet (HFD)-induced diabetic mouse model, we showed for the first time that downregulation of active β-catenin due to abnormal GSK3β activation caused synaptic neurodegeneration of RGCs by inhibiting ROS scavenging enzymes, thus triggering oxidative stress-driven mitochondrial impairment in HFD-induced diabetes. Rescue of β-catenin via ectopic expression of β-catenin with a recombinant adenoviral vector, or via GSK3β inhibition by a targeted si-*GSK3β*, through intravitreal administration, abrogated the oxidative stress-derived mitochondrial defect and synaptic neurodegeneration in diabetic RGCs. By contrast, ablation of β-catenin by si-*β*-*catenin* abolished the protective effect of GSK3β inhibition on diabetic RGCs by suppression of antioxidant scavengers and augmentation of oxidative stress-driven mitochondrial lesion. Thus, our data identify β-catenin as a part of an endogenous protective system in diabetic RGCs and a promising target to develop intervention strategies that protect RGCs from neurodegeneration at early onset of diabetic retinopathy.

## INTRODUCTION

Diabetic retinopathy (DR) remains a leading cause of vision loss in adults world-wide and has long been classified as a microvascular complication of diabetes [1]. However, increasing amounts of evidence emphasize that neurodegeneration of retinae is a critical and early component of DR, which even precedes the development of detectable microvascular signs of DR [2–4]. Of particular note, synaptic neurodegeneration of retinal ganglion cells (RGCs) occurs before its apoptosis and appears as the earliest step in the pathogenesis of diabetic retinal neurodegeneration [5–7]. Thus, characterization of key molecular events involved in RGCs synaptic degeneration is imperative for preventing diabetic retinal neurodegeneration.

Mitochondrial dysfunction is considered as the prime factor responsible for degeneration and loss of neurons in the pathogenesis of neurodegenerative diseases [8]. Maintenance of normal structure and function of synapse requires ATP demands provided by mitochondria [9, 10]. We have recently shown that aberrant hyperphosphorylation of the microtubule-associated protein tau inhibits microtubule-dependent axonal/dendritic trafficking of mitochondria, thereby depriving synapses of mitochondria and causing the starvation of synapses, leading to synapse loss and dysfunction of RGCs in diabetic retinae [6]. Thus, our previous study proposes that impairment of mitochondrial trafficking by hyperphosphorylated tau is a potential contributor to diabetic RGCs synapse degeneration [6]. However, other molecular mechanisms underlying mitochondrial defect in diabetic retinal neurodegeneration remain to be elucidated.

Mitochondrion is a major site of reactive oxygen species (ROS) production, and also a target for destructive effect of oxidants, indicating the presence of a vicious cycle of oxidative damage [8, 11]. Recent studies have shown that oxidative stress due to the chronic accumulation of ROS is the significant instigator of hyperglycemia- or dyslipidaemia-induced abnormal mitochondrial functions in diabetic retinal vascular endothelial cells [12–14]. Inhibition of antioxidant ROS scavengers, resulting in excess accumulation of ROS, is one of the oxidative stress-induced dysfunctions in mitochondria. Previous studies have reported that suppression of ROS scavenging enzymes, such as manganese superoxide dismutase (MnSOD) and glutathione peroxidase (GSH), were involved in oxidative damage of mitochondria in vascular endothelial cells from diabetic retinae [15, 16]. Nevertheless, the role, if any, and the molecular mechanism of oxidative stress-driven mitochondria damage in diabetic RGCs synapse neurodegeneration have yet to be ascertained.

The canonical Wnt/β-catenin signaling has been implicated in the maintenance of development and normal function of specific retinal neurons [17] as well as in the protection of RGCs from neurodegeneration both *in vitro* and *in vivo* [18–20]. Phosphorylation of β-catenin at residues of serine 33, serine 37 and threonine 41 (Ser33/37/Thr41) by GSK3β, a major component of the destruction complex, is a signal for the rapid proteasomal degradation of β-catenin [21, 22]. In response to Wnt or PI3K/Akt signals, inactivation of GSK3β through phosphorylation at Ser9 [23] leads to the stabilization, cytosolic accumulation and subsequent translocation of β-catenin to the nucleus, where β-catenin acts as a transcriptional activator, transactivating the expression of specific genes together with Lef1/Tcf transcription factors [24]. Of interest, β-catenin has been shown to directly regulate the transcription of genes encoding a few of antioxidant scavengers, such as glutathione peroxidase 2 (GPx2), catalase (CAT), thioredoxin reductases TrxR2 and TrxR3 in response to ROS [25–28]. Loss of β-catenin fails to suppress the generation of ROS, thus triggering oxidative stress and leading to injury of hepatocyte survival and hematopoietic regeneration [28, 29], indicating the importance of β-catenin in antagonizing oxidative stress. However, little is known about the role of β-catenin in oxidative stress-driven mitochondria damage in the context of diabetic RGCs synapse degeneration.

The aim of the present study was firstly to explore the putative roles of oxidative stress-derived mitochondria damage in diabetic RGCs synapse neurodegeneration in a well-established high-fat diet (HFD)-induced diabetic mouse model, where degeneration of retinal neural cells precedes the development of visible retinal microvascular lesions as we and others reported [30–33]. In addition, the changes and potential roles of β-catenin in HFD-induced diabetic RGCs neurodegeneration, as well as the regulation of β-catenin signaling in oxidative stress-driven mitochondria damage were further investigated.

## RESULTS

### Mitochondrial impairment is associated with HFD-induced diabetic retinal neurodegeneration

**Figure 1:**
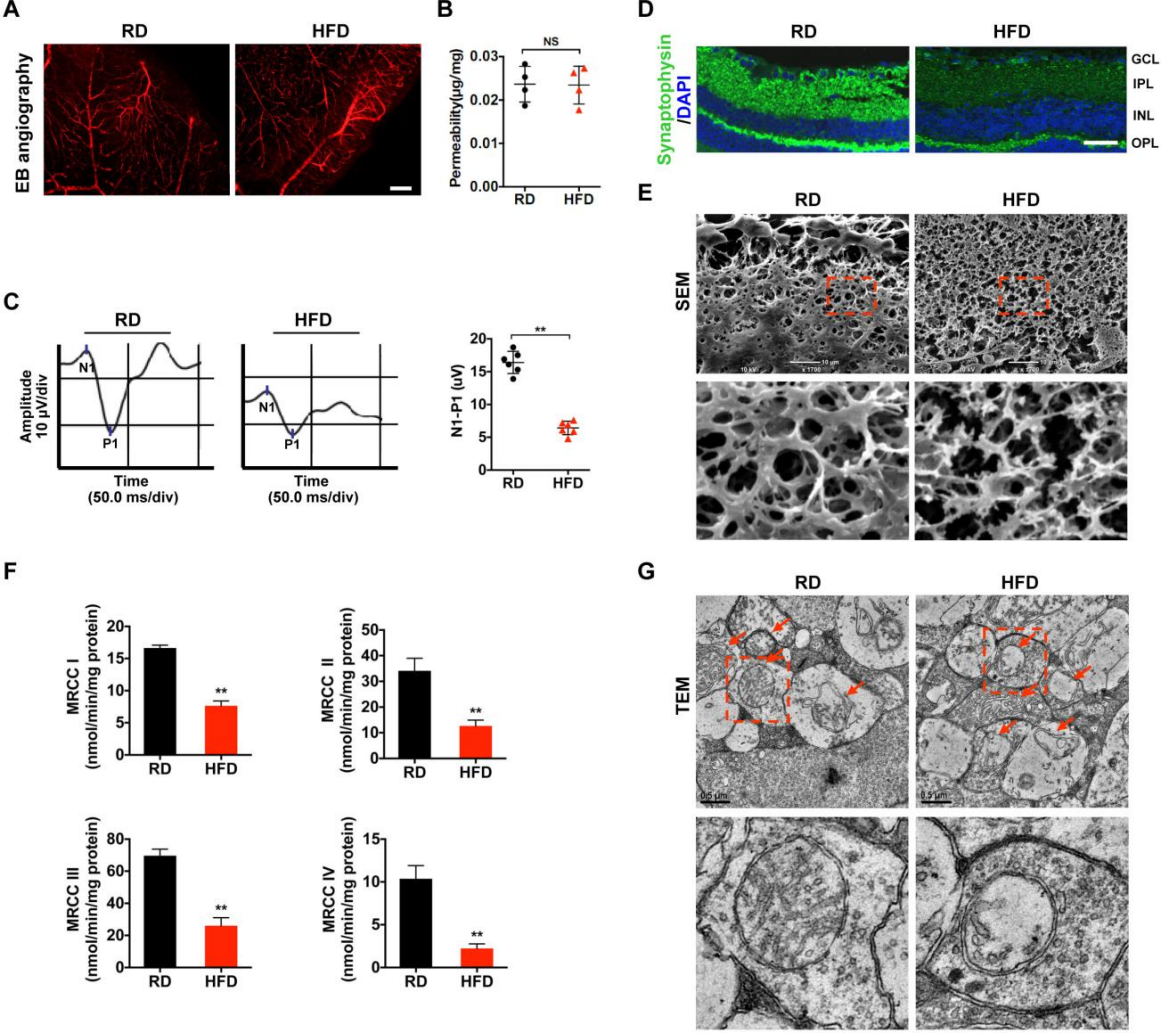
**Mitochondrial impairment is associated with HFD-induced diabetic retinal neurodegeneration.** (**A**) Representative images of retinal Evans Blue (EB) angiography from mice fed with regular chow (RD) or HFD for 20 weeks. Scale bar, 150 μm. (**B**) Retinal vascular leakage was quantified and normalized by total retinal protein concentrations, and expressed as μg of EB per mg of proteins. NS, no significant difference. (**C**) Representative waveforms of visual evoked potential (VEP). The differences in peak amplitude (N1-P1) were quantified. (**D**) Representative retinal immunofluorescence staining for synaptophysin (green). Nuclei were counterstained with DAPI (blue). Scale bar, 100 μm. GCL, ganglion cell layer; IPL, inner plexiform layer; INL, inner nuclear layer; OPL, outer plexiform layer. (**E**) Representative scanning electron microscopy (SEM) of retinal sections. Lower panels are high-power magnification of the areas indicated by the boxes. Scale bar, 10 μm. (**F**) Activities of retinal mitochondrial complex I-IV (MRCC I-IV) were measured by spectrophotometry and expressed as nmol/min/mg protein. Two retinae from 2 respective mice in the same group were pooled and preceded for each experiment. Three independent experiments were performed in duplicate for each group. (**G**) Representative transmission electron microscopy (TEM) of neural retinal sections. Mitochondria in IPL are indicated with arrows. Lower panels are high-power magnification of the areas indicated by the boxes. Scale bar, 0.5 μm. Data are means ± SEM. n = 4 (**A**, **B**) or n = 6 (**C**–**G**) mice per group. ^**^P < 0.01 vs age-match RD controls. See also Supplementary Figure 1; Supplementary Figures 2A and 3A.

Our previous study showed that HFD for 20 weeks can elicit diabetic RGCs dysfunction and synapse degeneration in the absence of detectable retinal microvascular abnormality [6, 33]. Therefore, in the current study, a 20-week HFD feeding regimen was used to set up a diabetes model (Supplementary Figure 1A–1G) with early retinal neurodegeneration. Whereas HFD for 20 weeks failed to trigger increased permeability of retinal vessels as determined by EB dye leakage assay (Figure 1A, 1B), it did significantly impair the electrophysiological function of RGCs as revealed by visual evoked potential (VEP) (Figure 1C), in comparison with age-matched RD controls. We further found that the compromised visual response of RGCs may be attributed to synaptic loss (Figure 1D; Supplementary Figure 2A) and dendritic disconnection (Figure 1E; Supplementary Figure 3A) in HFD-induced diabetic RGCs, as revealed by immunofluorescence staining of synaptophysin, a vesicular protein regulating presynaptic neurotransmitter, and scanning electron microscopy (SEM) of retinal sections, respectively. Of particular note, disturbance of mitochondrial function, as demonstrated by reduced activity of mitochondrial complex I-IV in the electron transport chain (Figure 1F), and disruption of mitochondrial structure, including mitochondrial swelling, vacuolization, and disappearance of mitochondrial cristae as determined by transmission electron microscope (TEM) of retinal sections (Figure 1G), were found to occur in HFD-induced diabetic retinae, indicating that mitochondrial impairment is associated with, and even be the prime factor responsible for diabetic retinal neurodegeneration.

### Oxidative stress causes mitochondrial damage and synapse degeneration in diabetic retinae

**Figure 2:**
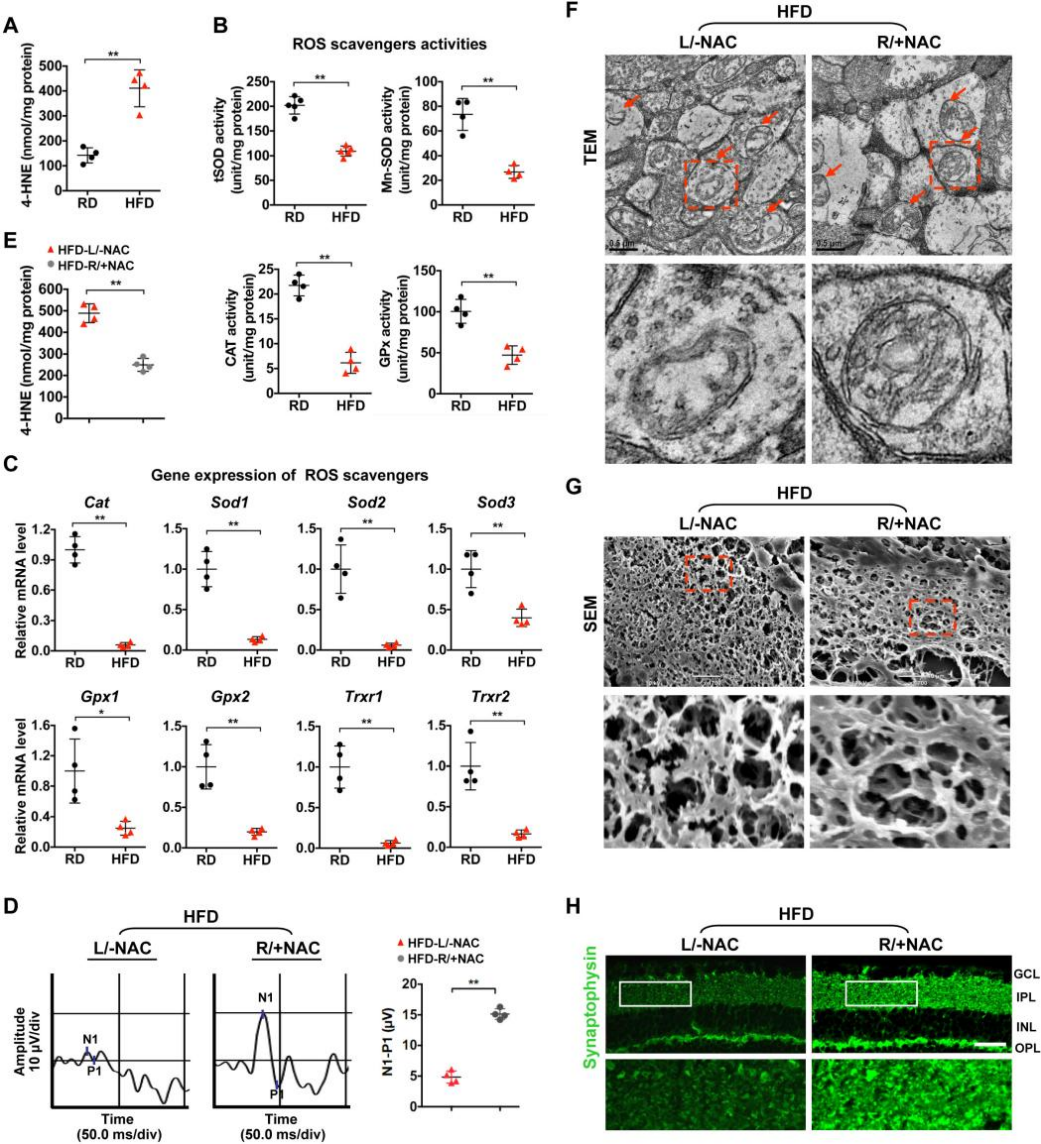
**Oxidative stress causes mitochondrial defect and synaptic neurodegeneration in diabetic retinae.** (**A**) Contents of 4-HNE in retinae. (**B**) Activities of antioxidant enzymes. (**C**) Expression of genes encoding ROS scavengers was determined by quantitative RT-PCR in retinae. The mRNA level of each gene was normalized to the internal GAPDH control and expressed as fold changes of mRNA abundance in the retina from HFD groups relative to their age-matched RD controls. (**D**–**H**) 1 μl of NAC (500 nM) was injected intravitreally into the right eye of HFD-induced diabetic mice (HFD-R/+NAC), while PBS was injected into the contralateral left eye as a control (HFD-L/-NAC). (**D**) Representative waveforms of VEP and quantification of differences in peak amplitude (N1-P1). (**E**) Contents of retinal 4-HNE. (**F**) Representative images of retinal TEM with mitochondria in IPL (arrows). Areas boxed in are shown at higher magnification in the lower panels. Scale bar, 0.5 μm. (**G**) Representative SEM of retinal sections. Areas boxed are shown at higher magnification. Scale bar, 10 μm. (**H**) Representative synaptophysin (green; scale bar, 100 μm) immunostaining in retinae. Areas boxed in are shown at higher magnification in the lower panels. Data are means ± SEM. n = 4 mice (**A**–**C**) or n = 4 eyes (**D**–**H**) per group. ^**^P < 0.01 vs age-match RD controls; ^*^P < 0.05 and ^**^P < 0.01 vs contralateral eye injected with PBS. See also Supplementary Figures 2B and 3B.

Given that increased oxidative stress plays a major role in mitochondrial dysfunction [13, 14], we set out to investigate whether oxidative stress contributes to mitochondrial impairment and synaptic neurodegeneration in HFD-induced diabetic retinae. We found an elevated level of 4-hydroxynonenal (4-HNE), a major oxidation product of membrane lipids that is considered an indicator of oxidative stress, in HFD-induced diabetic retinae as relative to that in age-matched RD controls (Figure 2A), suggesting that enhanced oxidative stress is associated with HFD-induced diabetic retinal neurodegeneration. As impaired removal of reactive oxygen species (ROS) leads to its abnormal accumulation, we then examined whether inhibition of antioxidant scavengers occurs in the pathogenesis of diabetic retinae. A significant decrease in the activity of antioxidant enzymes (Figure 2B), including catalase (CAT), total superoxide dismutase (tSOD), manganese superoxide dismutase (MnSOD), and glutathione peroxidase (GPx), as well as reduced mRNA levels of genes encoding antioxidant scavengers, including *Cat*, *Sod1*, *Sod2*, *Sod3*, *Gpx1*, *Gpx2*, *Trxr1*, and *Trxr2* (Figure 2C), were detected in HFD-induced diabetic retinae, in comparison with RD controls, suggesting that the antioxidant defense was jeopardized in diabetic retinae.

To further confirm whether inhibition of ROS scavenging enzymes causes increased oxidative stress-associated mitochondrial lesion and retinal neurodegeneration, 1 μl of N-acetyl-L-cysteine (NAC, 500 nM), a generic ROS scavenger, was injected intravitreally into the right eye of HFD-induced diabetic mice, while PBS was injected into the contralateral left eye as a control [34]. Forty-eight hours after intravitreal administration, a significantly improved visual response of RGCs was observed in NAC-treated eyes of HFD mice as compared to vehicle-treated eyes (Figure 2D), which was attributed to the decrease of 4-HNE production in diabetic retinae (Figure 2E), thus leading to rescue of mitochondrial structure (Figure 2F), dendritic connection (Figure 2G; Supplementary Figure 3B) and synaptophysin expression (Figure 2H; Supplementary Figure 2B) upon NAC treatment. These data suggest that enhanced oxidative stress due to reduction of antioxidant scavengers causes mitochondrial damage and synapse degeneration of retinae in HFD-induced diabetic mice.

### Downregulation of β-catenin contributes to oxidative stress-induced mitochondrial damage and synapse degeneration in diabetic retinae

**Figure 3:**
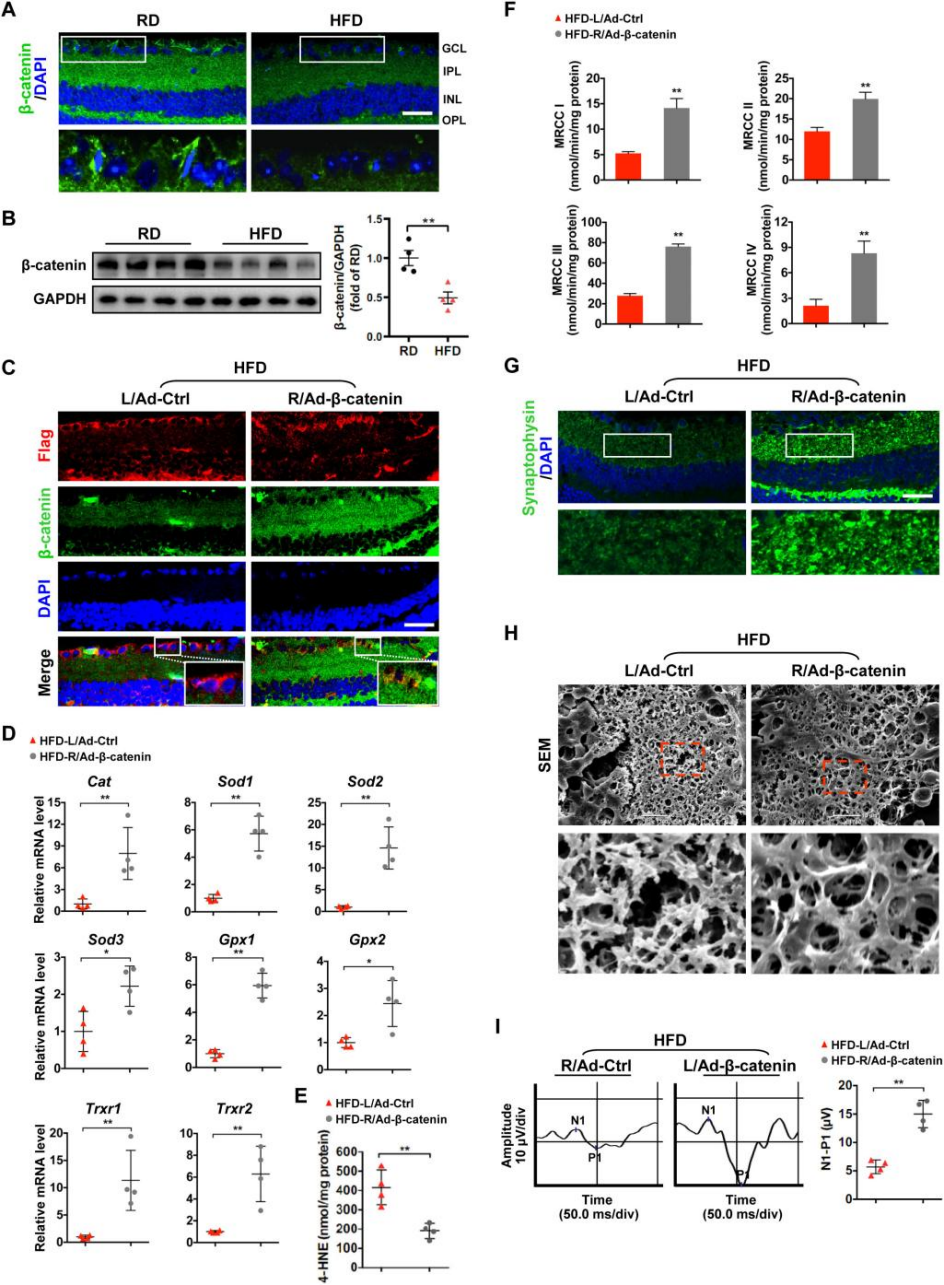
**β-catenin downregulation triggers oxidative stress-induced mitochondrial damage and synapse degeneration in diabetic retinae.** (**A**) Representative retinal immunofluorescence for active β-catenin (green) from mice fed with RD or HFD. Nuclei were counterstained with DAPI (blue). Areas boxed in are shown at higher magnification in the lower panels. Scale bar, 100 μm. (**B**) Western blotting of active β-catenin in total retina lysate. Intensities were quantified and normalized against the level of GAPDH and expressed as fold changes of protein abundance in the retina from HFD groups relative to RD controls. (**C**–**I**) An adenovirus coding for β-catenin with Flag tag was injected intravitreally into the right eye of HFD-fed mice (HFD-R/Ad-β-catenin), while an empty control vector was injected into the contralateral left eye as a control (HFD-L/Ad-Ctrl). (**C**) Retinal double-immunostaining for Flag (red) and active β-catenin (green). Areas boxed in are shown at higher magnification. Scale bar, 100 μm. (**D**) Relative mRNA expression of genes encoding ROS scavengers. (**E**) Contents of 4-HNE in retinae. (**F**) Activities of retinal mitochondrial complex MRCC I-IV. Two retinae from 2 respective eyes in one group were pooled. Three independent experiments were performed in duplicate for each group. (**G**) Representative retinal immunostaining for synaptophysin (green; scale bar, 100 μm). Areas boxed in are shown at higher magnification in the lower panels. (**H**) Representative SEM of retinal sections. Areas boxed are shown at higher magnification. Scale bar, 10 μm. (**I**) Representative VEP waveforms and quantification of peak amplitude difference (N1-P1). Data are means ± SEM. n = 4 mice (**A**–**B**), n = 4 eyes (**C**–**E**; **G**–**I**), or n = 6 eyes (**F**) per group. ^**^P < 0.01 vs age-match RD controls; ^*^P < 0.05 and ^**^P < 0.01 vs contralateral eye injected with Ad-Ctrl. See also Supplementary Figures 2C, 3C, 4A, and 4B.

Given that transcription activator β-catenin has been reported to regulate the expression of antioxidant scavengers in response to ROS [25, 27, 28], we investigated whether the increased oxidative stress due to reduced ROS scavengers is associated with the defect of β-catenin in diabetic retinae. We found that the expression of active (non-phosphorylation at Ser33/37/Thr41) and total β-catenin was significantly downregulated in HFD-induced diabetic neural retinae, as compared to age-matched RD controls (Figure 3A, 3B; Supplementary Figure 4A; Supplementary Figure 5). To further examine whether restoration of β-catenin can rescue the compromised antioxidant defense-related diabetic retinal neurodegeneration, a recombinant adenovirus coding for β-catenin with N-terminal Flag tag (Ad-β-catenin) was injected intravitreally into the right eye of HFD-fed mice, while an empty control vector (Ad-Ctrl) was injected into the contralateral left eye as a control. A comparable infection efficiency was achieved between Ad-β-catenin (~70%) and Ad-Ctrl (~72%) one week after intravitreal administration as revealed by immunostaining of Flag (Figure 3C). Strikingly, rescue of β-catenin expression in neural retinae (Figure 3C; Supplementary Figure 4B) significantly increased the transcriptional expression of antioxidant scavengers (Figure 3D), hence leading to decrease in 4-HNE accumulation (Figure 3E) and increase in mitochondrial activity (Figure 3F), synaptophysin expression (Figure 3G; Supplementary Figure 2C), terminal dendritic complexity (Figure 3H; Supplementary Figure 3C), and visual response of RGCs (Figure 3I) in Ad-β-catenin-treated eyes of HFD-induced diabetic mice, in comparison with contralateral Ad-Ctrl-treated eyes. Collectively, these data demonstrate that downregulation of β-catenin results in reduced expression of ROS scavengers and increased intracellular oxidative stress, which further triggers mitochondrial impairment and retinal neurodegeneration in HFD-induced diabetes.

### Rescue of β-catenin by GSK3β inhibition protects diabetic retinae from oxidative stress-induced mitochondrial impairment and synapse neurodegeneration

**Figure 4:**
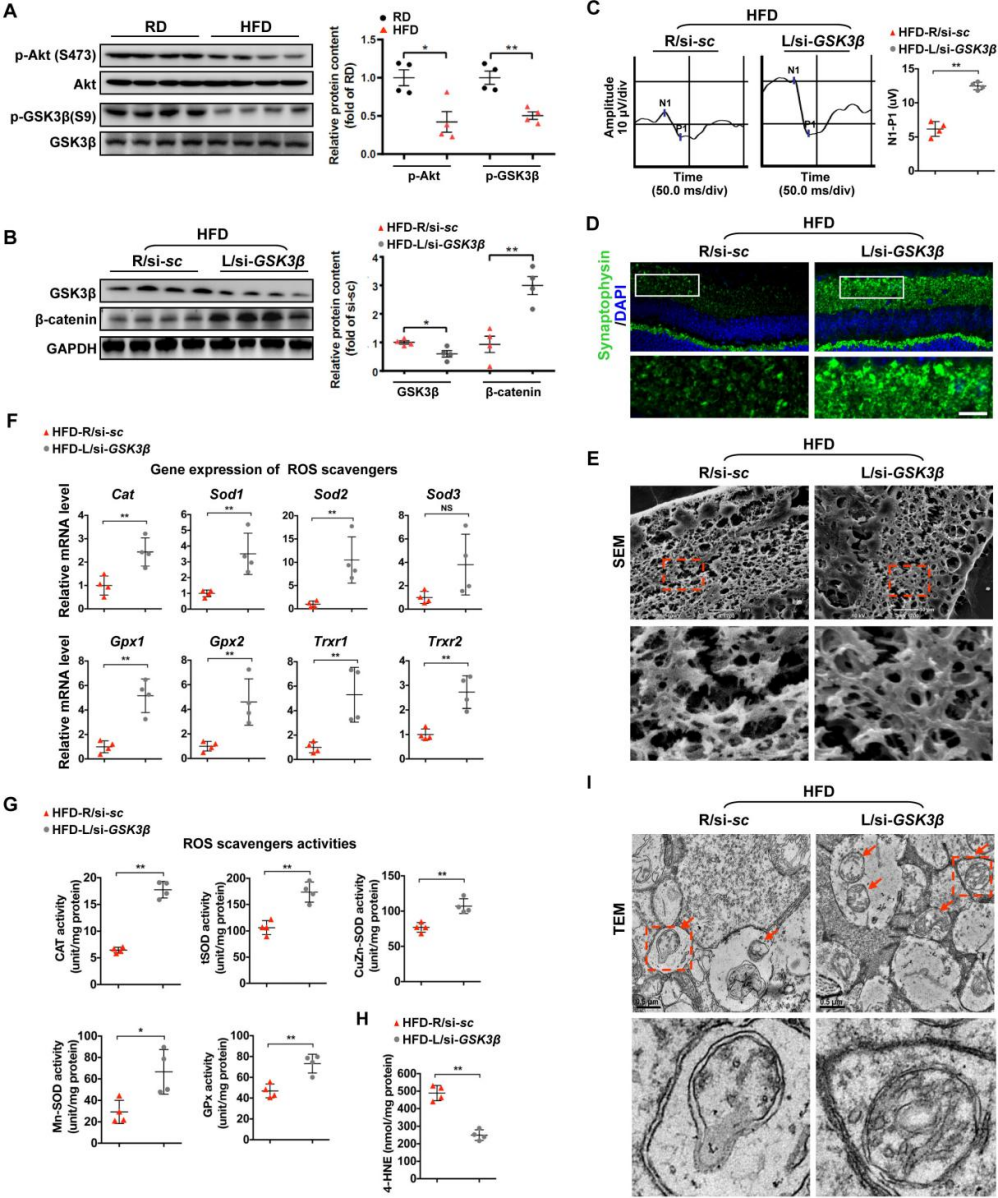
**Restoring β-catenin by GSK3β inhibition protects diabetic retinae from mitochondrial and synaptic defect.** (**A**) Western blotting analyses of phosphorylated-Akt (S473), Akt, phosphorylated-GSK3β (S9), and GSK3β in retinae from mice fed with RD or HFD, respectively. Relative intensities were quantified and normalized against the level of Akt or GSK GSK3β, respectively. (**B**–**I**) si-*GSK3β* was intravitreally injected in the left eye of HFD-fed mice (HFD-L/si-*GSK3β*), while a scramble si-sc was injected in the contralateral right eye as a control (HFD-R/si-sc). (**B**) Western blotting for GSK3β and active β-catenin. Relative intensities were quantified. (**C**) Representative waveforms of VEP and quantification of differences in peak amplitude (N1-P1). (**D**) Retinal immunostaining for synaptophysin (green; scale bar, 100 μm). Areas boxed in are shown at higher magnification. (**E**) Representative images of retinal SEM. Lower panels are high-power magnification of boxed areas. Scale bar, 10 μm. (**F**) Relative mRNA expression of ROS scavenging genes in retinae. (**G**) Activities of antioxidant enzymes in retinae. (**H**) Contents of retinal 4-HNE. (**I**) Representative images of retinal TEM with mitochondria in IPL (arrows). Areas boxed in are shown at higher magnification. Scale bar, 0.5 μm. Data are means ± SEM. n = 4 mice (**A**) or n = 4 eyes (**B**–**I**) per group. ^*^P < 0.05 and ^**^P < 0.01 vs age-match RD controls; ^*^P < 0.05 and ^**^P < 0.01 vs contralateral eye injected with scramble si-*sc*. NS, no significant difference. See also Supplementary Figures 2D and 3D.

We then sought to investigate the regulation of β-catenin, a protein that is primarily targeted for proteasomal degradation by direct phosphorylation at Ser33/37/Thr41 by GSK3β. Interestingly, our previous studies showed that HFD can induce dysregulation of Akt/GSK3β signaling, including abnormal inactivation of Akt and activation of GSK3β in diabetic retinae [6, 33]. In consistent with our previous work, we found a significant decrease in activating phosphorylation of Akt (Ser473) and inactivating phosphorylation of GSK3β (Ser9) in retinae from mice fed a HFD (Figure 4A). To further examine the role of GSK3β in HFD-induced diabetic retinal neurodegeneration, a siRNA targeted to GSK3β (si-*GSK3β*) was intravitreally injected in mice at 20 weeks after HFD, while scramble si-*sc* was injected in the contralateral eye as a control. Remarkably, knock-down of GSK3β in diabetic retinae (Figure 4B) considerably ameliorated HFD-induced diabetic retinal neurodegeneration, including improving VEP responses (Figure 4C), synaptophysin expression (Figure 4D; Supplementary Figure 2D), and connection of dendritic arbors (Figure 4E; Supplementary Figure 3D) one week after intravitreous administration. The protective effect of GSK3β inhibition was mostly likely attributed to its upregulation of protein levels of active β-catenin (Figure 4B), thus leading to increased transcriptional expression (Figure 4F) and activities (Figure 4G) of ROS scavengers, reduced accumulation of the oxidative stress products, 4-HNE (Figure 4H), and improved structure of mitochondria (Figure 4I).

### β-catenin is required for the protective effect of GSK3β inhibition against diabetic retinal neurodegeneration

**Figure 5:**
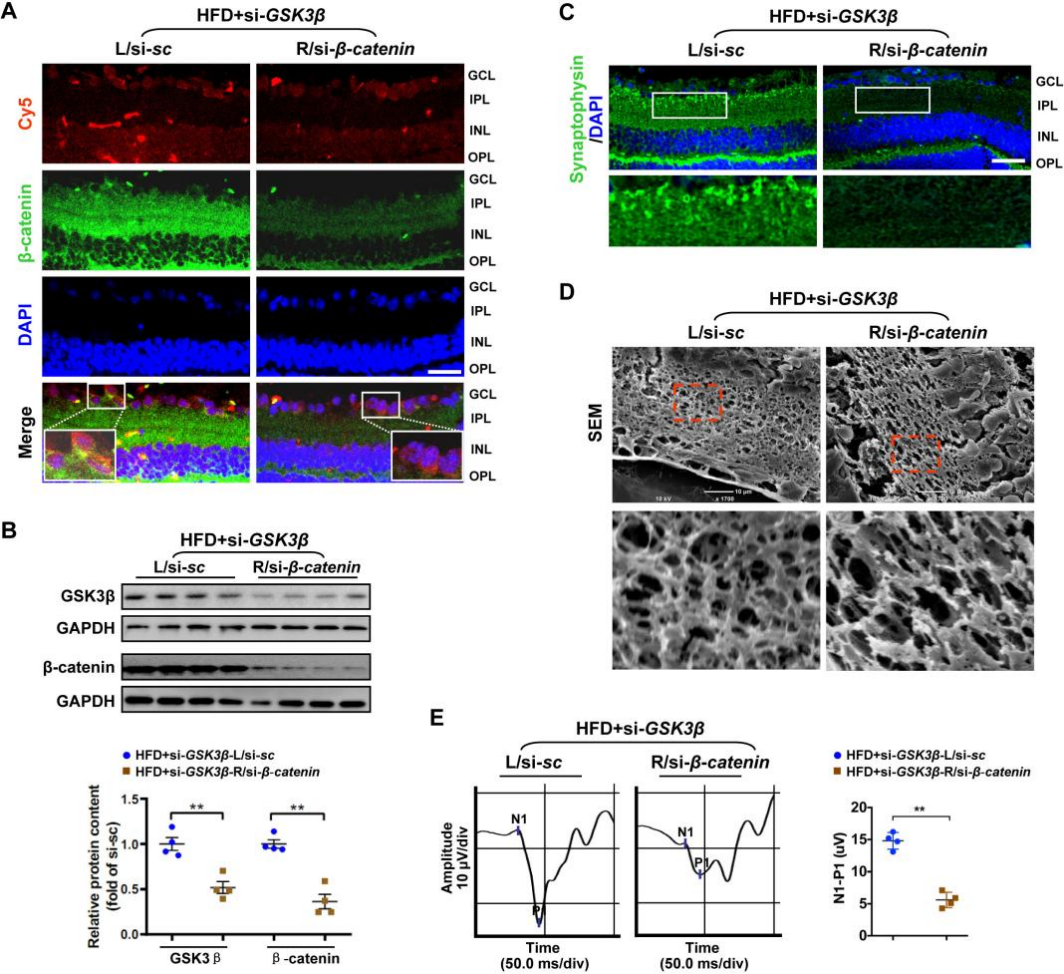
**Knock-down of β-catenin abrogated the protective effect of GSK3β depletion on HFD-induced diabetic retinal neurodegeneration.** A Cy5-labeled si-*β-catenin* was intravitreally co-injected with si-*GSK3β* into the right eye of HFD-induced diabetic mice (HFD+si- *GSK3β*/R-si-*β-catenin*), while scramble si-*sc* was co-administrated with si-*GSK3β* in the contralateral left eye as a control (HFD+si-*GSK3β*/L-si-*sc*). (**A**) Representative immunofluorescence for active β-catenin (green, active β-catenin; red, Cy5; blue, DAPI; scale bar, 100 μm). Areas boxed in are shown at higher magnification. (**B**) Western blotting for GSK3β and active β-catenin. Relative intensities were quantified. (**C**) Representative retinal immunofluorescence staining for synaptophysin (green; scale bar, 100 μm). Areas boxed in are shown at higher magnification. (**D**) Representative SEM of retinal sections. Areas boxed are shown at higher magnification. Scale bar, 10 μm. (**E**) Representative waveforms of VEP and quantification of differences in peak amplitude (N1-P1). Data are means ± SEM. n = 4 eyes per group. ^**^P < 0.01 vs contralateral eye injected with scramble si-*sc*. See also Supplementary Figures 2E, 3E and 4C.

**Figure 6:**
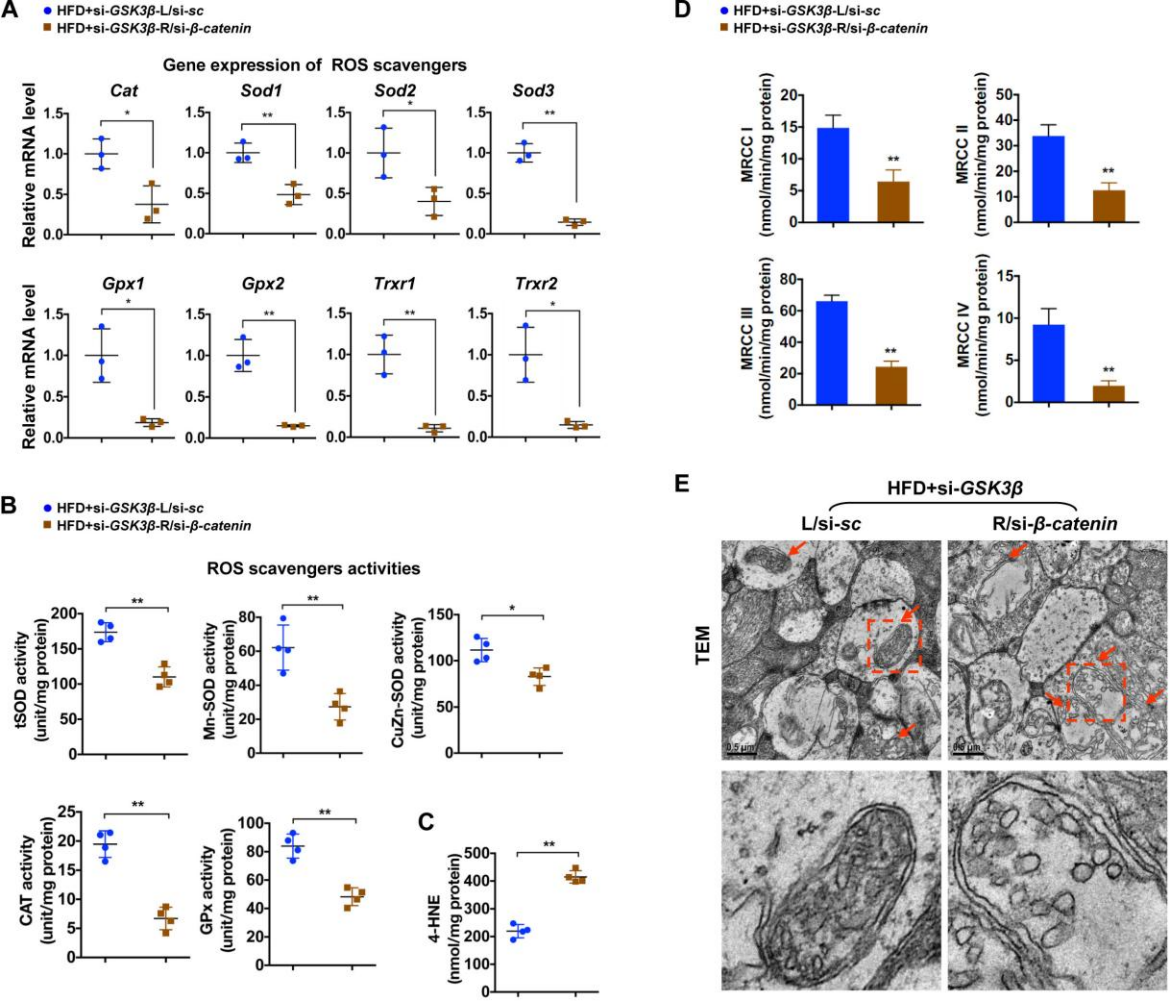
**β-catenin is required for effects of GSK3β inhibition against oxidative stress-induced mitochondrial damage.** (**A**) Relative mRNA expression of ROS scavenging genes in retinae from eyes treated with si-*GSK3β*+si-*β-catenin* (HFD+si-*GSK3β*/R-si-*β-catenin*) or si-*GSK3β*+si-*sc* (HFD+si-*GSK3β*/L-si-*sc*), respectively. (**B**) Activities of antioxidant enzymes in retinae. (**C**) Amounts of retinal 4-HNE. (**D**) Activities of retinal mitochondrial complex MRCC I-IV. Two retinae from 2 respective eyes in one group were pooled. Three independent experiments were performed in duplicate for each group. (**E**) Representative images of retinal TEM with mitochondria in IPL indicated with arrows. Areas boxed in are shown at higher magnification in lower panels. Scale bar, 0.5 μm. Data are means ± SEM. n = 4 eyes (**A**–**C**; **E**) or n = 6 eyes (**D**) per group. ^*^P < 0.05 and ^**^P < 0.01 vs contralateral eye injected with scramble si-*sc*.

To test whether rescue of β-catenin is critical for the beneficial effect of GSK3β suppression on diabetic retinae, a Cy5-labeled siRNA targeted to β-catenin (si-*β-catenin*) was intravitreally co-administrated with si-*GSK3β* into the right eye of HFD-induced diabetic mice, while scramble si-*sc* was co-injected with si-*GSK3β* in the contralateral eye as a control. As relative to scramble control, knock-down of β-catenin (Figure 5A, 5B; Supplementary Figure 4C) abrogated the protective effect of GSK3β depletion on HFD-induced diabetic neural retinae, leading to a significant decrease in synaptophysin expression (Figure 5C; Supplementary Figure 2E), terminal dendritic connection (Figure 5D; Supplementary Figure 3E), and visual response of RGCs (Figure 5E) in diabetic retinae a week after intravitreous co-administration. The abolished beneficial effect of GSK3β inhibition on protecting diabetic retinae from neurodegeneration when β-catenin was knocked down was owing to the reduced transcriptional expression (Figure 6A) and activities (Figure 6B) of β-catenin-targeting ROS scavengers, thus resulting in increased production of 4-HNE (Figure 6C) and impairment of mitochondria (Figure 6D, 6E). Taken together, these data indicate that β-catenin is requisite for the protective effect of GSK3β inhibition against oxidative stress-induced mitochondrial damage and synapse neurodegeneration in diabetic retinae.

### Inhibition of GSK3β prevents oxidative stress-associated mitochondrial dysfunction and synaptic loss of primary RGCs upon glucolipotoxicity via activation of β-catenin

**Figure 7:**
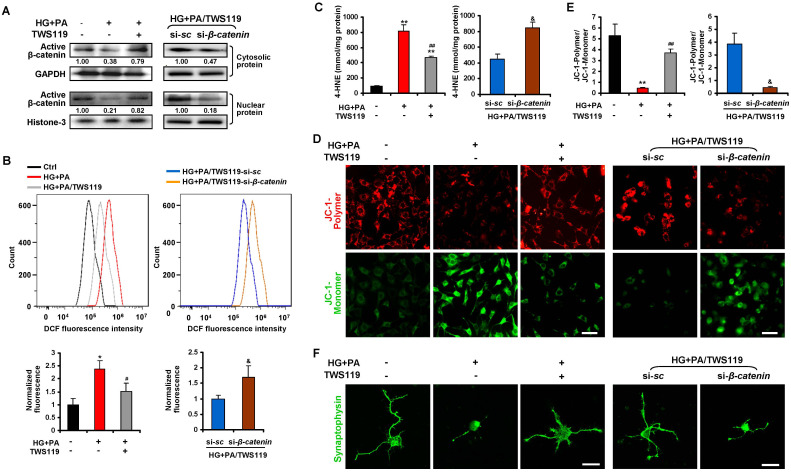
**Dysregulated GSK3β/β-catenin signaling caused oxidative stress-associated mitochondrial and synaptic damage of primary RGCs upon glucolipotoxicity.** Primary RGCs were exposed to conditioned medium (HG+PA) for 24 h, in the absence or presence of TWS119. Alternatively, RGCs were transfected by si-*β-catenin* or si-*sc* and treated with HG+PA in the presence of TWS119. (**A**) Western blotting for active β-catenin in cytosolic and nuclear fraction of primary RGCs. Intensities were quantified and normalized against the level of GAPDH or Histone-3 and expressed as fold changes of protein abundance relative to controls. Relative intensities of the bands are shown below. (**B**) Intracellular ROS production was measured by a flow cytometer. Representative curvilineal profiles of fluorescence are shown in upper panels. Quantification of intracellular ROS is shown in lower panels. Values are expressed as the fold changes relative to controls. (**C**) Contents of 4-HNE in primary RGCs. (**D**) The mitochondrial membrane potential (MMP) was determined with JC-1 using a confocal microscope. Representative images are shown (green, JC-1 monomer; red, JC-1 polymer; scale bar, 50 μm). (**E**) The ratio of red to green fluorescence intensity which reflects the levels of the MMP was quantified using Image-Pro Plus software. (**F**) Representative synaptophysin (green; scale bar, 20 μm) immunostaining in primary RGCs. Data are means ± SEM of three independent experiments. ^*^P < 0.05 and ^**^P < 0.01 vs normal control; ^#^*P* < 0.05 and ^##^*P* < 0.01 vs HG+PA; ^&^*P* < 0.05 vs si-*sc*. See also Supplementary Figures 6, 8A and 8B.

Considering the diversity of cell types in neural retinae, we further evaluated the role of GSK3β/β-catenin in diabetic RGCs synaptic degeneration *in vitro*. Chronic exposure of cultured primary neonatal rat RGCs (Supplementary Figure 6) to 10 mM of D-glucose together with 200 μM of BSA-conjugated palmitate was adopted to generate a model of HFD-induced glucolipotoxicity *in vitro* as previously described [6]. In line with the *in vivo* findings, there was a considerable decrease in inactivating phosphorylation of GSK3β (Ser9), cytosolic and nuclear levels of active β-catenin, and transcriptional activity of β-catenin in RGCs upon glucolipotoxicity (Figure 7A; Supplementary Figure 7; Supplementary Figure 8A, 8B), accompanied by a remarkable increase in production of ROS (Figure 7B) and 4-HNE (Figure 7C), and a decrease in mitochondrial membrane potential (Figure 7D, 7E) and synaptophysin expression (Figure 7F), as compared to control cells. Inactivation of GSK3β by a specific GSK3β inhibitor, TWS119 (10 nM), significantly restored the expression and transcriptional activity of β-catenin (Figure 7A; Supplementary Figure 8B), leading to inhibition of oxidative stress (Figure 7B, 7C), mitochondrial dysfunction (Figure 7D, 7E) and synaptic loss (Figure 7F) in glucolipotoxicity-challenged RGCs. However, simultaneous knock-down of β-catenin and GSK3β in RGCs abrogated the protective effects of GSK3β inhibition upon glucolipotoxicity (Figure 7A–7F). Thus, our data demonstrate that inhibition of GSK3β protects primary RGCs from oxidative stress-associated mitochondrial dysfunction and synaptic loss upon glucolipotoxicity via activation of β-catenin.

## DISCUSSION

Identification of key molecular events involved in synaptic neurodegeneration of RGCs, the earliest step in the pathogenesis of diabetic retinal neurodegeneration [5–7], is vital for arresting disease development. The present study demonstrates for the first time that loss of β-catenin due to GSK3β activation triggers RGCs synaptic neurodegeneration by inducing oxidative stress-driven mitochondrial damage in HFD-induced diabetes.

Our previous study showed that impairment of microtubule-dependent axonal/dendritic trafficking of mitochondria to distal synapses by abnormal tau hyperphosphorylation caused synapse loss and dysfunction of RGCs in HFD-induced diabetes [6], indicating that defects in mitochondrial transport is an important contributor to diabetic RGCs synaptic neurodegeneration. In the current study, we found that the structure and function of mitochondria was also jeopardized in HFD-induced diabetic retinae and glucolipotoxicity-stressed primary RGCs, emphasizing that mitochondrial impairment may be the prime factor responsible for diabetic retinal neurodegeneration. Although several lines of evidence have proposed that oxidative stress is the significant initiator of mitochondrial dysfunction in diabetic retinal vascular endothelial cells [12, 35], the role of oxidative stress in mitochondrial impairment-associated diabetic RGCs neurodegeneration remains far to be clarified. Here, we showed for the first time that enhanced oxidative stress due to suppression of antioxidant ROS scavengers contributed to mitochondrial defect and synaptic degeneration in diabetic retinae, since intravitreally administration of a generic ROS scavenger, NAC, remarkably alleviated the lesion of mitochondria and the neurodegenerative phenotype of diabetic RGCs.

Of interest, recent studies have suggested that inhibition of ROS accumulation by antioxidants such as MnSOD mimics represents a possible direction for mitochondria-targeted treatment in the retinal microvascular disorder of DR [13, 36]. However, it is not known whether this treatment strategy is also beneficial in the retinal neurodegeneration at the early onset of DR. Furthermore, it is worthwhile to note that antioxidants supplement has been reported to have the potential to blunt a glucose-triggered ROS signaling, thereby inhibiting glucose-stimulated insulin secretion from pancreatic β-cells at the early stage of diabetes, which might increase the likelihood of exacerbating diabetic conditions [37, 38]. To avoid the possible destruction of pancreatic β-cell function by systemic administration of antioxidant, a strategy of intravitreal injection of the generic ROS scavenger NAC was adopted in the present work. Our data thus propose lowering oxidative stress by induction of ROS scavengers in diabetic RGCs as an appropriate alternative to improve mitochondrial function and arrest diabetic retinal neurodegeneration.

Despite that Wnt/β-catenin signaling is reported to protect neural cells from degeneration [18–20], it remains unexplored whether β-catenin signaling provides diabetic RGCs protection against oxidative stress. The data we report here attribute a key function of β-catenin in controlling oxidative stress resistance through transcriptional regulation of antioxidant scavengers in the context of HFD-induced diabetic retinal neurodegeneration. We found that suppression of ROS scavengers was at least partially accredited to a significant loss of active β-catenin in HFD-induced diabetic neural retinae. Although no convincing nuclear immunostaining of β-catenin was demonstrated in the ganglion cell layer of retinae *in vivo*, probably due to the abundant amounts of β-catenin at cell membranes and synapses of neural cells, or the dynamics of β-catenin activity which requires a time-resolved analysis, thereby rendering the detection of nuclear β-catenin particularly challenging as previous reported in the brain [39, 40], a considerable decrease in nuclear localization and expression of β-catenin was readily detected in glucolipotoxicity-stressed primary RGCs *in vitro*. More importantly, we further showed that ectopic expression of β-catenin in diabetic neural retinae by intravitreal administration of a recombinant Ad-β-catenin vector significantly rescued the synaptic neurodegeneration of diabetic RGCs by induction of endogenous ROS scavengers and thus inhibition of oxidative stress and mitochondrial impairment. By contrast, ablation of *β-catenin* via intravitreous injection of si-β-catenin abolished the protective effect of GSK3β inhibition on diabetic RGCs by suppression of antioxidant scavengers and augmentation of oxidative stress-driven mitochondrial lesion. Thus, our gain- and loss- of-function studies unravel a causative role of β-catenin in resisting oxidative stress-driven mitochondrial damage and synaptic neurodegeneration of diabetic RGCs. Interestingly, activation of Wnt/β-catenin signaling were found to contribute to the generation of ROS in diabetic retinal capillary endothelial cells [41, 42]. It is thus reasonable to speculate that the neuroprotective role of β-catenin in retinal neural cells appears to act largely independently from its effects on retinal vascular endothelial cells in the context of DR with as yet unidentified mechanisms. In such case, a cell-type (retinal neuron or capillary endothelial cells) specific targeting of β-catenin would be highly desirable to avoid the off-target effect when considering β-catenin as a therapeutic target of drug development for different pathogenic process in DR.

Another intriguing finding in this work was the observation that abnormal activation of GSK3β contributed to the loss of active β-catenin, since inhibition of GSK3β by a targeted siRNA or a specific inhibitor significantly restored the protein levels of active β-catenin and abrogated the oxidative stress-derived mitochondrial defect and synaptic degeneration in HFD-induced diabetic retinae or glucolipotoxicity-stressed primary RGCs, respectively. Of interest, GSK3β acts not only as a major component of the destruction complex for β-catenin, but also a predominant kinase for tau phosphorylation. Our previous study [6] has demonstrated that activation of GSK3β triggers abnormal tau hyperphosphorylation, which leads to destabilization of microtubules and impairment of microtubule-dependent transport of mitochondria to distal synapses. Collectively, our previous and present studies establish abnormal GSK3β activation as an important contributor to mitochondrial defect in diabetic retinal neurodegeneration, not only by triggering hyperphosphorylated tau-induced impairment in synaptic targeting of mitochondria [6], but also by inhibiting β-catenin-regulated oxidative stress resistance. Indeed, the specific GSK3β inhibitor has emerged as a therapeutic target for drug development for tau pathology in neurodegenerative disease [43], our studies thus broaden the theoretical basis for the treatment of neurodegenerative diseases with GSK3β inhibitors.

**Figure 8:**
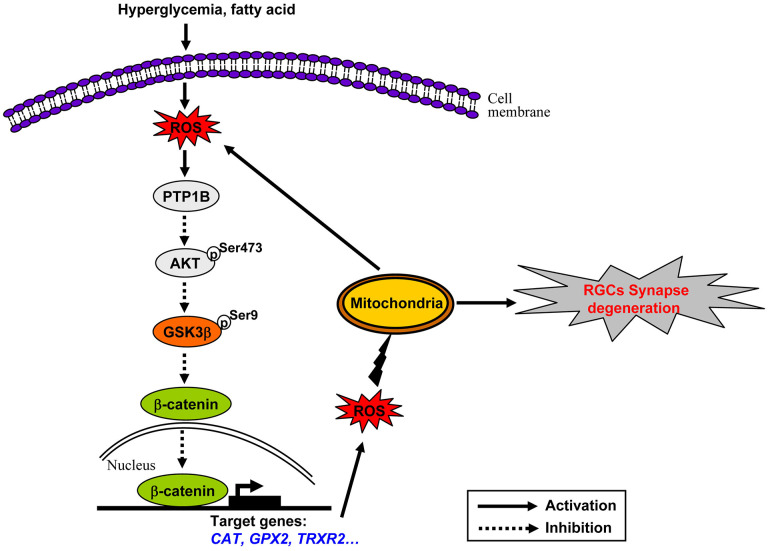
**Schematic of molecular mechanisms underlying oxidative stress-driven mitochondrial impairment in HFD-induced diabetic retinal neurodegeneration.** See the text for a detailed description.

GSK3β activation is a downstream effecter of dysregulated insulin signaling in HFD-induced diabetic brain and retina as we and others have reported [6, 33, 44, 45]. Of note, deficiency of insulin signaling under HFD feeding conditions has been shown to be resulted from increased expression of protein tyrosine phosphatase 1B (PTP1B) [46, 47], a key negative regulator of insulin signaling [48], which can interact with insulin receptor (IR) and insulin receptor substrate (IRS-1) to hydrolyze tyrosine phosphorylation induced by insulin signals [49]. In this study, we also found a significant increase in the protein expression of PTP1B in HFD-induced diabetic retinae (Supplementary Figure 9), suggesting that activation of PTP1B is implicated in the inhibition of IRS/Akt signaling in this context. Intriguingly, recent studies have shown that increased PTP1B expression is induced by activation of ROS-NF-κB axis [50–52]. NF-κB is an important signal transduction molecule that has been recognized as a downstream target of ROS [53], which, once activated by ROS, is able to bind to the promoter of *PTP1B* and induces its expression [50–52]. Of note, activation of NF-κB pathway in response to ROS was recognized as an early event in HFD-induced insulin resistance, which occurred after 3 weeks of HFD feeding [53]. Therefore, we envisage the following scenarios for chronic oxidative stress-driven mitochondrial impairment in HFD-induced diabetic retinal neurodegeneration (Figure 8). Increased PTP1B is an early response to dyslipidaemia-induced ROS production through mitochondria, which then instigates the dysregulation of insulin signaling, thus sequentially leading to activation of GSK3β, downregulation of active β-catenin, inhibition of endogenous ROS scavengers, and persistent accumulation of ROS. The chronically enhanced oxidative stress further causes the impairment of mitochondria, which on one hand leads to generation of more oxidants, thus rendering a vicious cycle of oxidative damage, on the other hand results in synapse neurodegenerations in HFD-induced diabetic RGCs.

## CONCLUSIONS

In summary, this study reports for the first time that β-catenin is a part of an endogenous protective system in RGCs, the loss of which due to abnormal GSK3β activation plays a causative role in instigating oxidative stress-driven mitochondrial damage during the pathogenesis of diabetic retinal neurodegeneration. β-catenin and the presence of the required cellular pathways to active β-catenin signaling appear to be promising targets to develop intervention strategies that protect RGCs from neurodegeneration at the early onset of DR.

## MATERIALS AND METHODS

### Reagents and antibodies

A recombinant adenovirus expressing β-catenin with N-terminal Flag tag (Ad-β-catenin) and an empty control (Ad-Ctrl) were purchased from Vigene Bioscience (Shandong, China). Short interfering RNA (siRNA) against β-catenin (mouse, sc-29210; rat, sc-270011) and their scramble controls (si-*sc*) was purchased from Santa Cruz Biotechnology (Santa Cruz, CA, USA); siRNA against GSK3β (si-*GSK3β*) 5’-CCACTCAAGAACTGTCAAGTA-3’ (sense strand) and scramble control (si-*sc*) 5’-UUCUCCGAACGUGUCACGUTT-3’ (sense strand) were ordered from Genemall (Shenzhen, China) [6]. N-acetyl-L-cysteine (NAC) was purchased from MedChemExpress (MCE, China). TWS119 was from Selleck (Trenton, NJ, USA). Palmitate and bovine serum albumin (BSA) was obtained from Sigma-Aldrich (St.Louis, MO, USA). 4’,6-diamidino-2-phenylindole (DAPI) was ordered from Invitrogen^TM^. Primary antibodies against non-phospho (active) β-catenin at Ser33/37/Thr41 (4270), phospho β-catenin at Ser33/37/Thr41 (9561), total β-catenin (9562), total Akt (4691), phosphorylated Akt at S473 (4060), total GSK3β (9315), phosphorylated GSK3β at S9 (9336), and GAPDH (2118) were ordered from Cell Signalling Technology (Boston, MA, USA). Antibodies against synaptophysin (ab32127), Thy1 (ab225), Tuj1 (ab18207), MAP2 (ab75713), PTP1B (ab189179), and histone H3 (ab1791) were purchased from Abcam (Cambridge, MA, USA). Primary mouse anti Flag tag antibody (AE005) was from ABclonal (Boston, MA, USA). Secondary antibodies, including Alexa-Fluor-488 goat anti-rabbit, -594 goat anti-mouse, and -405 goat anti-chicken IgG were from Invitrogen^TM^ (Life Technology, MA, USA). Normal donkey serum (ab7475) was purchased from Abcam (Cambridge, MA, USA).

### Animals

Male C57BL/6 mice were purchased from Guangdong Medical Laboratory Animal Center (Guangzhou, China). Starting at 5 weeks of age, mice were fed either a HFD (45% fat calories; Mediscience Ltd, China) or regular standard laboratory chow (RD) as controls (10% fat calories; Mediscience Ltd) as we previously described [6, 33] for 20 weeks. Their food intake (3-5 g of RD or HFD/mice/day) and body weight were monitored throughout the feeding regimen. Neonatal Sprague-Dawley rats (1-3 days old) used for isolation of primary RGCs were purchased from Guangdong Medical Laboratory Animal Center. All the animal experiments and maintenance were approved by the Laboratory Animal Ethics Committee of Shenzhen University.

### Metabolic parameters

Glucose or insulin tolerance tests were performed by an oral administration of D-glucose (1 g/kg) (OGTT) or an intraperitoneal injection of insulin (0.75 unit/kg, Gibco, Life Technologies, Grand Island, NY, USA) (IPITT) after an 8 h fast, respectively, as previously described [6]. Blood glucose levels were determined immediately before (0 min) and at 30, 60, 90, and 120 min after glucose or insulin administration from the tail vein with a Blood Glucose Meter (ACCU-CHEK Active, Roche, China). Levels of serum triglyceride (TG) and free fatty acids (FFA) were measured with a commercial kit (Wako Chemicals, Osaka, Japan). Levels of fasting serum insulin were determined with an Insulin (Mouse) Ultrasensitive ELISA kit (ALPCO Diagnostics, CA, USA).

### Visual evoked potential (VEP)

VEP was performed as described in our previous studies [6, 33]. In brief, after dark adaption and anesthesia, visual responses of mice were evoked with 300 consecutive flash stimuli and an intensity of 500 cd·sec/m^2^ for 2 min to one eye from a RETI-Port/Scan 21 recorder (Roland Consult, Wiesbaden, Germany),

### Retinal vascular permeability

Retinal vascular permeability was determined using Evans Blue (EB) as tracer. Briefly, mice were anesthetized and received a tail vein injection of EB dye (45 mg/kg of 1% solution, Sigma-Aldrich, CA, USA). Two hours later, a 0.2 ml of blood sample was collected from mice which were then perfused via the left ventricle with 1 ml warm PBS followed by 1% paraformaldehyde. The eyes were enucleated and retinae were dissected. For retinal EB angiography, retinae were flat-mounted and imaged at 620 nm with a Leica DMI4000B inverted fluorescence microscope (Leica Microsystems, Wetzlar, Germany). Alternatively, retinae and blood samples were treated with and without dimethylformamide (Sigma Aldrich, MO, USA), respectively. Extract supernatants were measured by a spectrophotometer at 620 nm and 740 nm, respectively. Retinal vascular permeability was expressed as microgram of EB per milligram of retinal proteins after normalization by concentrations of total retinal protein as previously described [54].

### Scanning electron microscopy (SEM)

SEM was performed as described in our previous studies [7, 33]. In brief, after fixation and dehydration, retinal cross sections were oriented and the exposed surface coated with gold-carbon vapor was examined by an electron microscope (JCM-6000, JEOL, Japan). Numbers of disconnected dendritic arbors were calculated for every image. Data from four independent sections were averaged for each eye.

### Transmission electron microscopy (TEM)

TEM was performed as previously described [34]. Briefly, dissected retinae were postfixed with a mixture containing 1% OsO4 and 0.8% potassium ferrocyanide in 0.1 M cacodylate buffer at 4 °C for 2 h, followed by dehydration and embedment in Epon. Ultrathin sections of retinae were then mounted on copper grids, stained with lead citrate and uranyl acetate, and examined under a LEO 906E electron microscope (Carl Zeiss, Oberkochen, Germany).

### Immunohistochemical analysis

Retinae and primary RGCs immunofluorescence analysis was performed as previously described [6, 33]. In brief, retinal sections or cells were incubated with primary antibodies (1:100 diluted in 1% Normal donkey serum in PBS) at 4 °C overnight, followed by incubating with secondary antibodies (1:100 in PBS) at 37 °C for 1 h. Nuclei were counterstained with DAPI. Images were obtained by a Carl Zeiss LSM880 Meta confocal microscope. Statistical quantitative analyses of retinal immunostaining were performed as previously described [33]. Briefly, the intensity of immunofluorescence staining was quantified by measuring the area of ganglion cell layer (GCL) and inner plexiform layer (IPL) under the curvilineal diagrams plotted with Origin 8.0 software. Data from three independent curvilineal diagrams were averaged for each eye.

### Western blot analysis

Whole retinae were lysed by sonication in 150 mM NaCl, 50 mM Tris (pH 7.4), 1 mM EDTA, 1% NP-40, 0.5% Triton X-100, and 2 mM NaVO3, in the presence of protease and phosphatase inhibitors. For detection of protein expression of β-catenin in the cytosol and nucleus, primary RGCs at a density of 1 x 10^7^ cells/dish were fractionated using the FractionPREP Cell Fractionation Kit (BioVision, Mountain View, CA, USA). Equal amounts of protein resolved by SDS-PAGE were immunoblotted as previously described [33] using primary antibodies listed above. Immunoreactive bands were revealed by enhanced chemiluminescence (SuperSignal™ West Pico Chemiluminescent Substrate kits, Thermo Scientific) and visualized by the KODAK Image Station 4000MM PRO. Band intensities were quantified by scanning densitometry (Gel-Doc2000, Bio-Rad) and analyzed with Quantity One™ (Bio-Rad).

### Mitochondrial respiratory chain complex activity

The activity of complex I-IV in mitochondrial electron respiratory chain was determined with the Micro Mitochondrial Respiratory Chain Complex I, II, III or IV Activity Assay Kit (Solarbio, Beijing, China) according to the manufacturer’s instructions. Briefly, two retinae freshly isolated from two respective mice in the same group were pooled and suspended in the mitochondrial complex extraction buffer, followed by gentle homogenization. The homogenate was centrifuged at 600 g for 10 minutes at 4 °C to remove cell debris and nuclei, and the supernatant was centrifuged again at 11,000 g for 15 minutes at 4 °C. The resultant pellet was resuspended in the extraction buffer and crushed by ultrasonication. Protein concentration of the extracted complex was determined by the bicinchoninic acid (BCA) assay (Sigma Chemical, St. Louis, MO). The complex activity of mitochondrial homogenates in the respective reaction buffer was then measured spectrophotometrically at 340 nm (complex I), 605 nm (complex II), and 550 nm (complex III and IV), respectively. Mitochondrial complex activity was expressed as nmol/min/mg protein. Three independent experiments were performed in duplicate for each group.

### ROS scavenging enzyme activity

In brief, freshly isolated retinae were homogenated and centrifuged at 1000 g for 20 min at 4 °C. The supernatant was quantitatively assessed using a BCA assay kit. The activity of catalase (CAT), total superoxide dismutase (tSOD), manganese superoxide dismutase (MnSOD), Copper/Zinc superoxide dismutase (CuZn-SOD), and glutathione peroxidase (GPx) was measured with the respective commercial assay kit (Nanjing Jianchen Bioengineering Institute, Nanjing, China) according to the the manufacturer’s instructions. Enzyme activity was normalized by total retinal protein concentrations and expressed as unit/mg protein.

### Determination of 4-HNE contents

Briefly, retinae or primary RGCs were homogenated and centrifuged at 1000 g for 20 min at 4 °C. Protein concentration in the supernatant was quantified by BCA assay. Contents of 4-HNE were determined with a 4 - hydroxy nonene aldehyde (4-HNE) ELISA assay kit from j&l Biological Industrial Co., Ltd. (Shanghai, China), normalized by total retinal protein concentrations, and expressed as nmol/mg protein.

### Quantitative real-time polymerase chain reaction (Q-PCR)

The RNA from retinae was extracted with the RNeasy Mini Kit (Qiagen, Germany). Reverse transcription was carried out using Random hexamers and SuperScript-III (Invitrogen). Quantitative real-time PCR was performed with the Applied Biosystems 7300 real-time systems using real-time PCR Master Mix (SYBR Green). The primer sequences are listed in Additional file 2 (Supplementary Table 1).

### Intravitreal injection

Intravitreal injections were performed as previously described [6, 55]. In brief, one microliter of the respective solution was slowly injected into the vitreous chamber of the eye from anesthetized mice through a Hamilton syringe fitted with a 30-gauge glass microneedle under a dissecting microscope. For ROS scavengers supplement, NAC (500 nM) [34] was injected intravitreally into the right eye and PBS was injected into the contralateral left eye, respectively. For rescue of β-catenin, an adenoviral vector Ad-β-catenin (5 x 10^7^ pfu/eye) was injected intravitreally into the right eye of HFD-fed mice, while an empty vector Ad-Ctrl was injected into the contralateral left eye as a control. For GSK3β depletion, si-*GSK3β* (2 μg/μl) was administrated into one eye and si-sc was applied into the contralateral eye. For co-repression of GSK3β and β-catenin, si-*GSK3β* (2 μg/μl) mixed with si-*β-catenin* (100 nM) or control si-*sc* was intravitreally injected into the right eye or contralateral left eye of HFD-fed mice, respectively.

### Isolation and treatment of primary RGCs

Primary RGCs were isolated from neonatal (1-3 days old) Sprague-Dawley rats as we previously described [6]. Cells after 5 or 6 days in culture were used for subsequent experiments. Identification of primary RGCs was performed by detecting the expression of RGC-characteristic marker Thy1, and neuronal markers Tuj1 and Map2 by immunofluorescence. Primary RGCs were treated with conditioned medium comprising 10 mM of D-gulcose and 200 μM of palmitate pre-conjugated to BSA or control medium containing 5 mM of D-glucose and 5 mM mannitol for 24 h, in the absence or presence of TWS119 (10 nM). With respect to β-catenin knock-down experiments, RGCs were transfected by si-*β-catenin* or si-*sc* with a Micropoly-transfecter^TM^ cell reagent (Biosky, China) according to the manufacturer’s instructions. Twenty-four hours after transfection, cells were subjected to conditioned medium in the presence of TWS119 for another 24 h.

### Measurement of intracellular ROS production

In brief, primary RGCs treated with different conditions were incubated with 10 μM DCFH-DA (Sigma-Aldrich), an indicator of general oxidative stress, for 1 h at 37 °C under 5% CO2. Cell suspensions were then centrifuged at 800 g for 5 min and washed with PBS twice. The fluorescence intensity was determined by a flow cytometer (Becton Dickinson, CA, USA) at excitation/emission of 485/528 nm, respectively. Three independent experiments were performed in duplicate for each condition.

### Mitochondrial membrane potential

The mitochondrial membrane potential (MMP) was determined with a fluorescent indicator JC-1 (Beyotime, China). Briefly, primary RGCs upon different treatment were incubated with 200 μM JC-1 for 20 min at 37 °C. JC-1 can enter the mitochondrial matrix to form polymer and generate red fluorescence. At low MMP, JC-1 exists in the form of monomer in the cytoplasm and yields green fluorescence. After washing twice with PBS to remove the remaining reagents, images were obtained with an OLYMPUS FV1000 confocal microscope. The immunufluorescence intensity was determined per cell in four consecutive fields that were randomly selected by Image-Pro Plus software. The ratio of red to green fluorescence intensity reflected the level of the MMP.

### Dual-luciferase reporter assay for transcription factor (TCF) binding

TOPFlash reporter or FOPFlash reporter (Millipore, Billerica, MA, USA) was co-transfected with an internal control Renilla luciferase reporter pRL-TK (Promega, Madison, WI, USA) into primary RGCs with the Micropoly-transfecter^TM^ cell reagent. Twenty-four hours after transfection, cells were subjected to the respective treatment for another 24 h. Cells were harvested and analyzed by the dual-luciferase assay kit (Promega, Madison, WI). Three independent experiments were performed in duplicate for each condition.

### Statistical analysis

Data were expressed as mean ± SEM. All statistical analyses were performed with GraphPad Prism 8.0 (GraphPad, San Diego, CA). Student’s *t*-test was used to analyze statistical significance between two groups. Statistically significant was considered when *P*<0.05.

### Ethics approval

Animals were handled in accordance with the Guide for the Care and Use of Laboratory Animals published by the US National Institutes of Health [(NIH) publication no. 85–23, revised 1996). All the animal experiments and maintenance were approved by the Laboratory Animal Ethics Committee of Shenzhen University (Permit Number: 201412042).

## Supplementary Material

Supplementary Figures

Supplementary Table 1
