# Supplementary material for: Loss of β-catenin via activated GSK3β causes diabetic retinal neurodegeneration by instigating a vicious cycle of oxidative stress-driven mitochondrial impairment
Source: Aging (Albany NY). 2020 Jun 23;12(13):13437–62. doi: 10.18632/aging.103446 (PMC7377872; doi:10.18632/aging.103446)
Supplement: Supplementary Figures [file aging-12-103446-s002..pdf]

SUPPLEMENTARY FIGURES

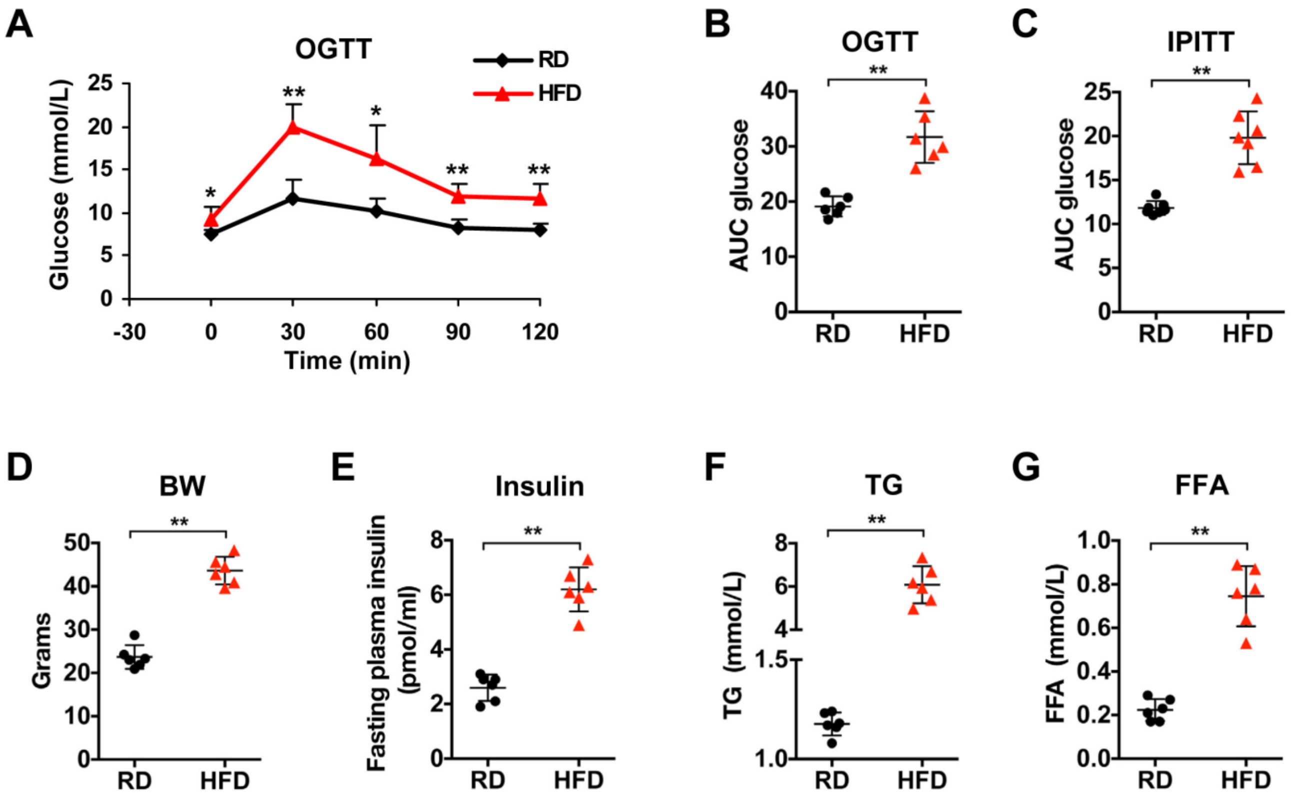

Supplementary Figure 1. Mice fed with HFD for 20 weeks developed features of type 2 diabetes. Mice were fed either a HFD providing 45% fat calories or regular chow (RD) for 20 weeks. (**A**) Oral glucose tolerance test (OGTT). (**B**) Calculation of the area under the curve (AUC) for OGTT. (**C**) Calculated AUC for intraperitoneal insulin tolerance tests (IPITT). (**D**) Body weight. (**E**) Fasting serum insulin. (**F**) Serum levels of triglycerides (TGG). (**G**) Serum levels of free fatty acids (FFA). Values are expressed as means ± SEM. n = 6 mice per group. $^{*}P < 0.05$ and $^{**}P < 0.01$ vs age-match RD controls.

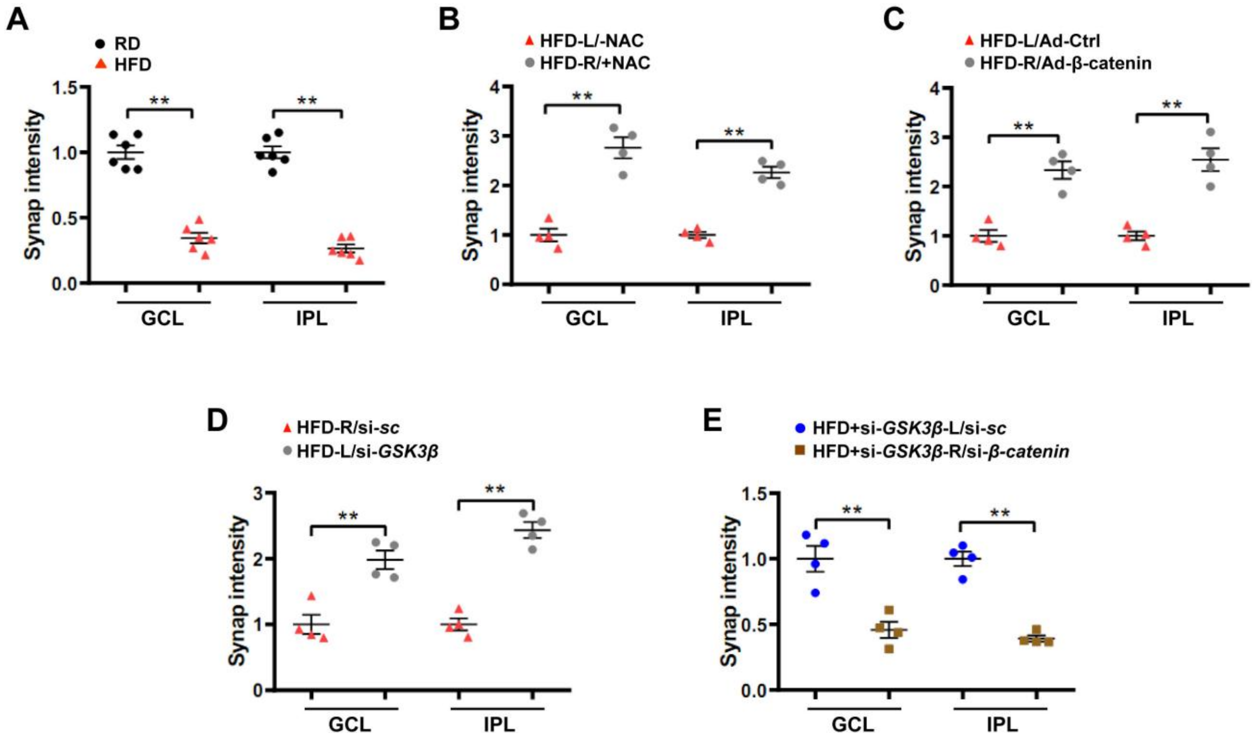

Supplementary Figure 2. Quantification of the immunostaining intensity of synaptophysin in retinae. The staining intensity of synaptophysin in GCL and IPL was quantified by measuring the AUC for the respective layer and expressed as relative staining intensity as compared to the respective control. Data are means ± SEM. n = 6 mice per group (**A**) or n = 4 eyes per group (**B**–**E**). $^{**}P < 0.01$ vs respective controls. Synap, synaptophysin; GCL, ganglion cell layer; IPL, inner plexiform layer.

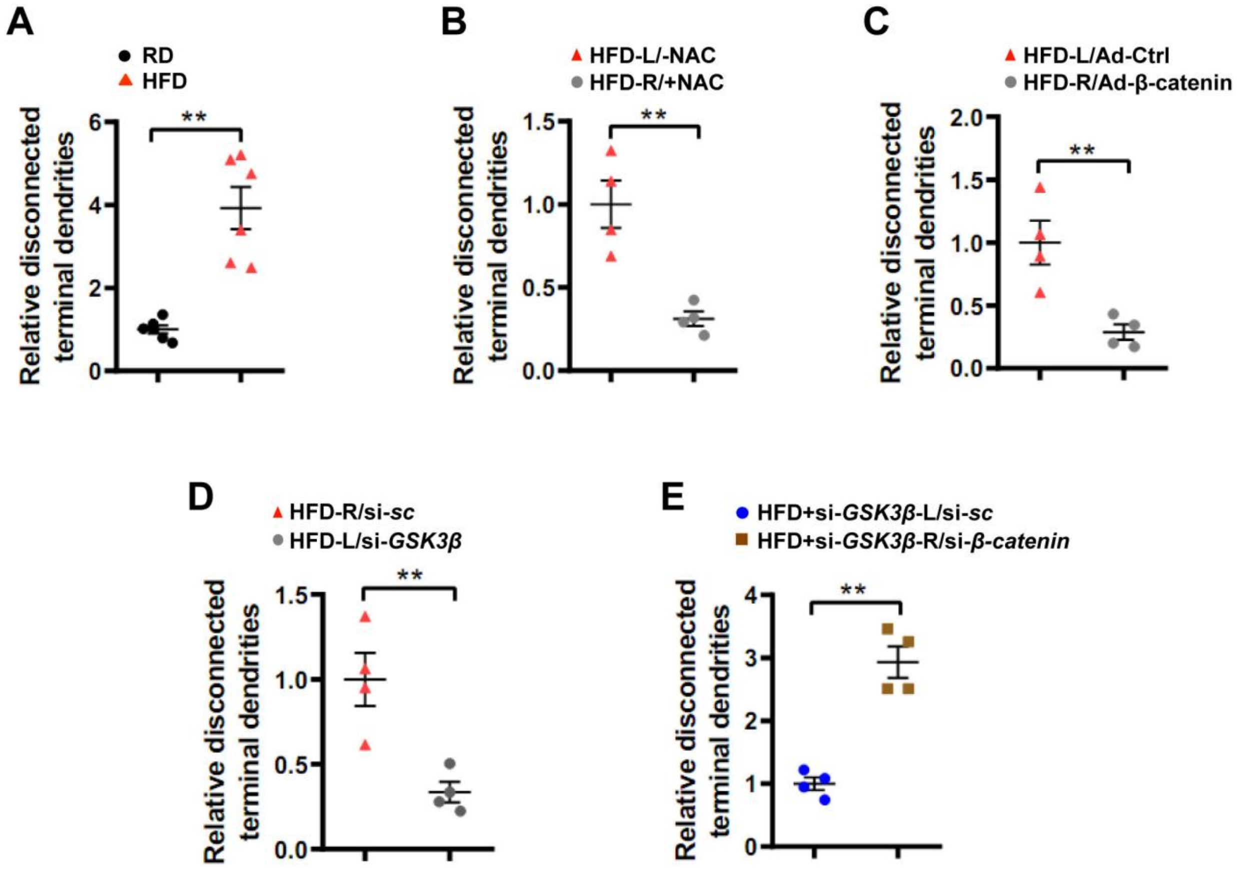

Supplementary Figure 3. Quantification of disconnected terminal dendrities determined by SEM. Numbers of terminal dendrities without connection in retinal SEM were quantified and expressed as fold changes of disconnected terminal dendrities in the retina relative to respective controls. Data are means ± SEM. n = 6 mice per group (**A**) or n = 4 eyes per group (**B**–**E**). ***P* < 0.01 vs respective controls.

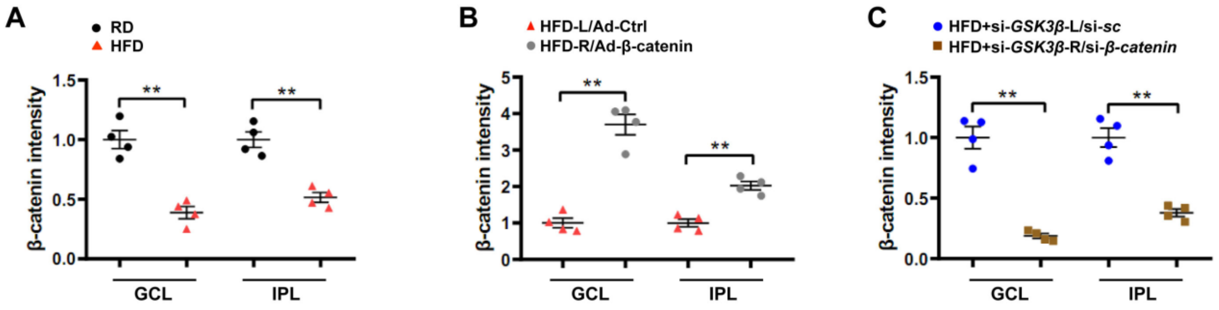

Supplementary Figure 4. Quantification of the immunostaining intensity of active β-catenin in retinae. The staining intensity of active β-catenin in GCL and IPL was quantified by measuring the AUC for the respective layer and expressed as relative staining intensity as compared to respective controls. Data are means ± SEM. n = 6 mice per group (**A**) or n = 4 eyes per group (**B**, **C**). ***P* < 0.01 vs respective controls.

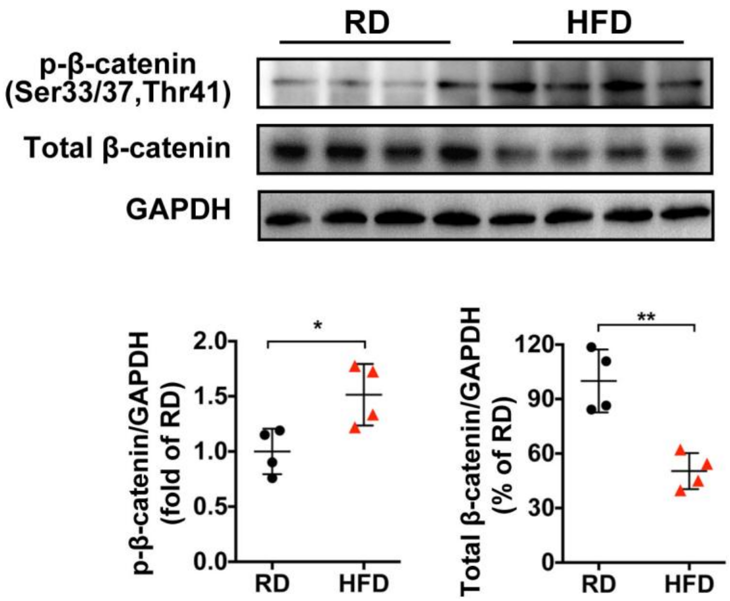

Supplementary Figure 5. Protein expression of phopho and total β-catenin. Western blot analyses of phospho- β-catenin (Ser33/37, Thr41) and total β-catenin in retinae from mice fed with RD or HFD for 20 weeks. Intensities were quantified and normalized against the level of GAPDH, and expressed as fold changes of protein abundance relative to RD. $^{*}P < 0.05$ and $^{**}P < 0.01$ vs age-match RD controls.

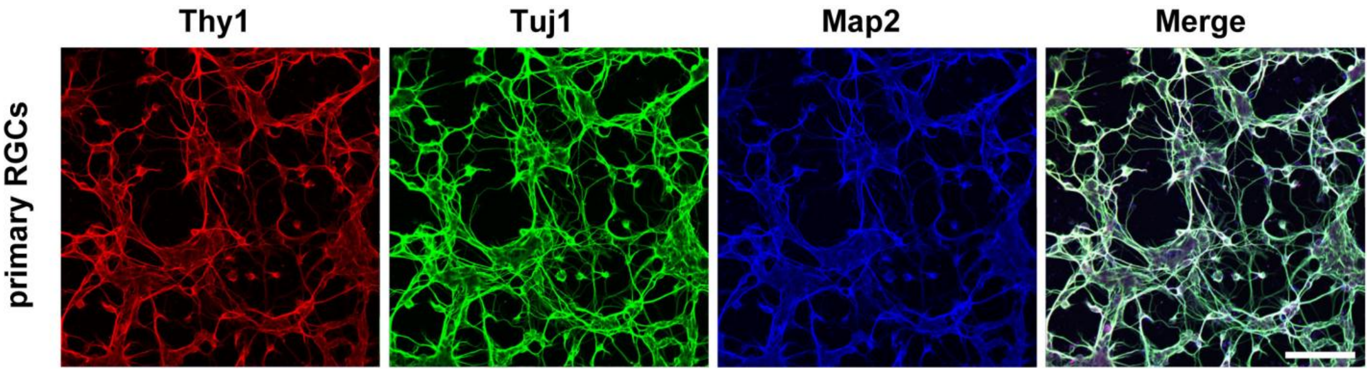

Supplementary Figure 6. Verification of primary RGCs. Representative images of triple immunostaining for RGC-characteristic marker Thy1 (red), neuronal markers Tuj1 (green) and Map2 (blue). Scale bar, 100 µm.

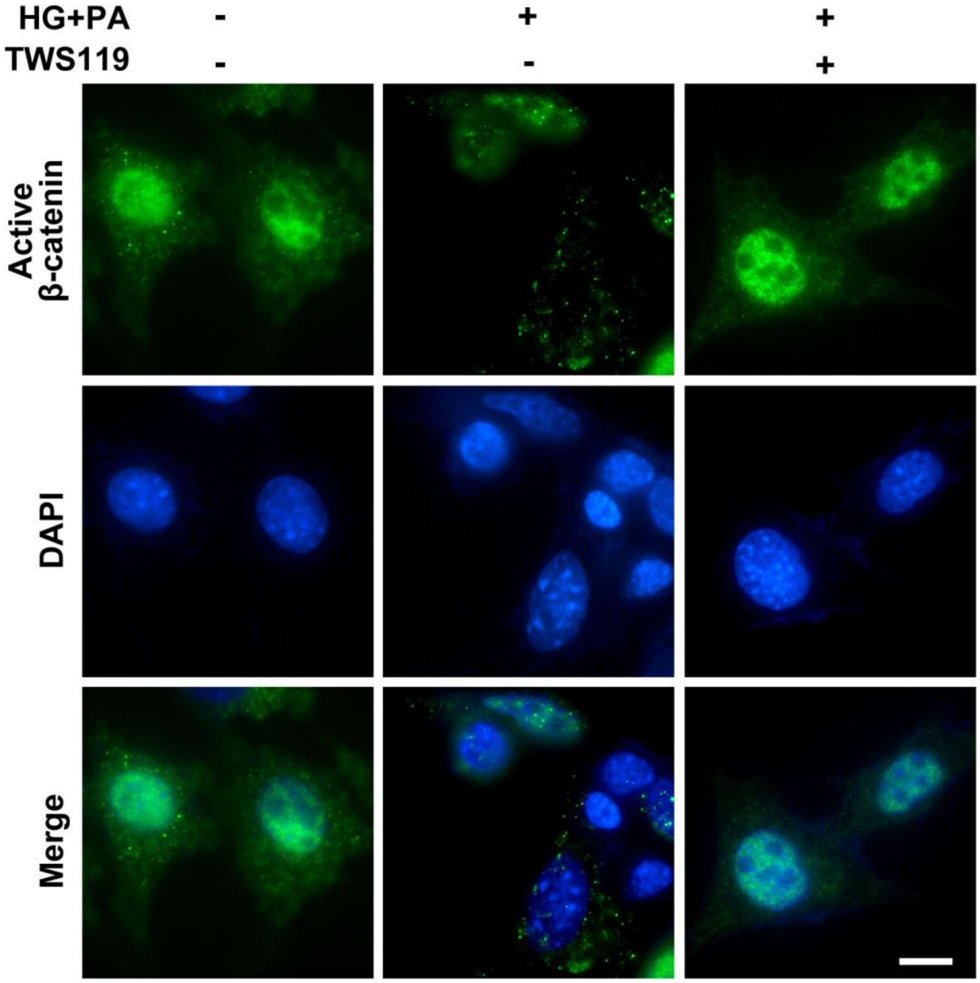

Supplementary Figure 7. Subcellular distribution of active β-catenin in RGCs. Immunofluorescence staining of active β-catenin (green) in RGCs exposed to conditioned (HG+PA) medium in the absence or presence of TWS119. Scale bar, 10 µm.

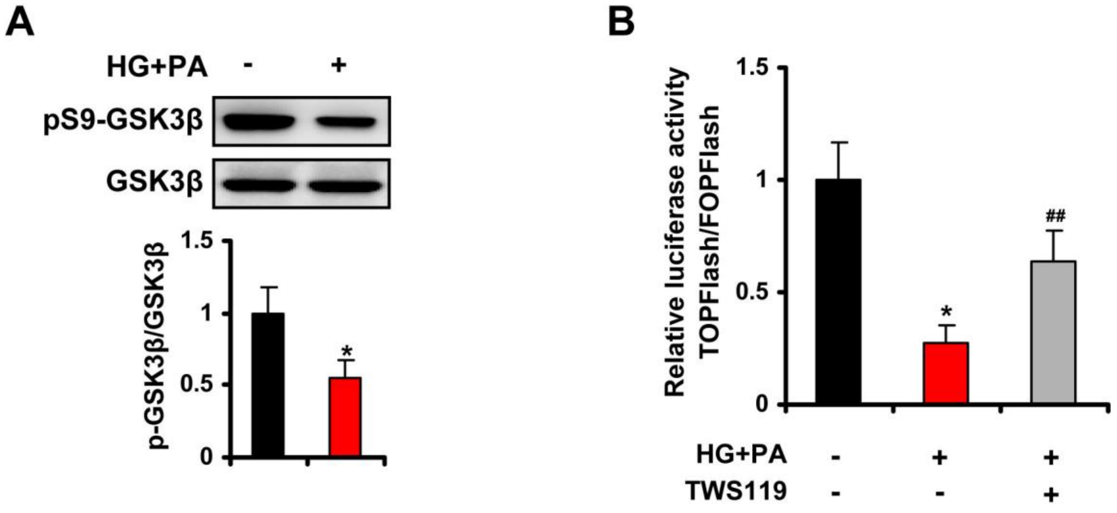

Supplementary Figure 8. Dysregulated GSK3β/β-catenin signaling in primary RGCs upon glucolipotoxicity stress. (**A**) Western blotting analyses for pGSK3β (Ser9) and GSK3β in primary RGCs exposed to control or conditioned (HG+PA) medium for 24 h, respectively. Intensities were quantified and normalized against the level of total GSK3β, and expressed as fold changes of protein abundance relative to normal controls. (**B**) Transcription activity of β-catenin was determined by TOPFlash luciferase reporter assay, with FOPFlash reporter as a control. Data are means ± SEM of three independent experiments. $^{*}P < 0.05$ vs normal control; $^{\#\#}P < 0.01$ vs HG+PA.

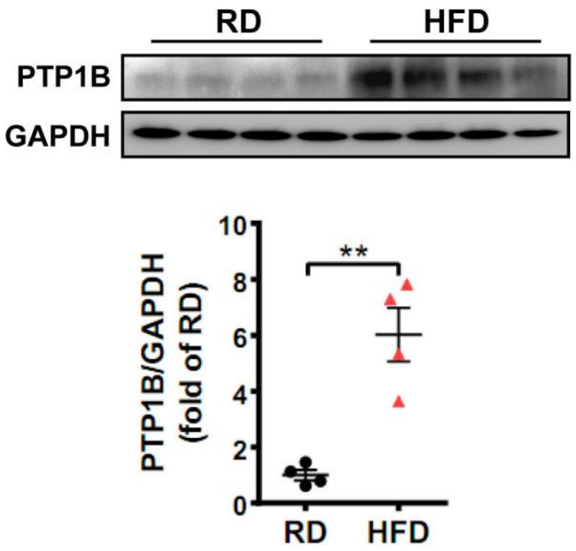

Supplementary Figure 9. Upregulation of PTP1B in HFD-induced diabetic retinae. Western blotting for PTP1B in retinae from mice fed with RD or HFD, respectively. Intensities were quantified and normalized against the level of GAPDH, and expressed as fold changes of protein abundance relative to RD controls. Data are means ± SEM. n = 4 mice per group. $^{**}P < 0.01$ vs RD control.
