# Supplementary material for: Loss of β-catenin via activated GSK3β causes diabetic retinal neurodegeneration by instigating a vicious cycle of oxidative stress-driven mitochondrial impairment
Source: Aging (Albany NY). 2020 Jun 23;12(13):13437–62. doi: 10.18632/aging.103446 (PMC7377872; doi:10.18632/aging.103446)
Supplement: Supplementary Table 1 [file aging-12-103446-s001..pdf]

SUPPLEMENTARY TABLE

Supplementary Table 1. Primer sequences for quantitative real-time PCR.

Gene		Sequence(5'-3')
Cat	Forward	GGAGGCGGGAACCCAATAG
	Reverse	GTGTGCCATCTCGTCAGTGAA
Sod1	Forward	AACCAGTTGTGTTGTCAGGAC
	Reverse	CCACCATGTTTCTTAGAGTGAGG
Sod2	Forward	CAGACCTGCCTTACGACTATGG
	Reverse	CTCGGTGGCGTTGAGATTGTT
Sod3	Forward	GGTTCCCAAATACTCTCTCTAAGG
	Reverse	CCCACCCCCAAGTTCCAT
Gpx1	Forward	CAGGAGAATGGCAAGAATGA
	Reverse	GAAGGTAAAGAGCGGGTGAG
Gpx2	Forward	ATCAAACGGCTCCTCAAAGT
	Reverse	GGGACGATATTCAGGGAATG
Trxr1	Forward	TCGTGGTGGACTTCTCTG
	Reverse	AGCAACATCCTGGCAGTCAT
Trxr2	Forward	GTTCCCCACATCTATGCCATTG
	Reverse	GGTTGAGGATTTCCCAAAGAGC
Gapdh	Forward	AGCAGTCCCGTACACTGGCAAAC
	Reverse	TCTGTGGTGATGTAAATGTCCTCT
